# Identification of neutral biochemical network models from time series data

**DOI:** 10.1186/1752-0509-3-47

**Published:** 2009-05-05

**Authors:** Marco Vilela, Susana Vinga, Marco A Grivet Mattoso Maia, Eberhard O Voit, Jonas S Almeida

**Affiliations:** 1 Instituto de Tecnologia Química e Biológica, Universidade Nova de Lisboa, Rua da Quinta Grande 6, Apartado 127, 2780-156 Oeiras, Portugal; 2 Instituto de Engenharia de Sistemas e Computadores: Investigação e Desenvolvimento (INESC-ID), R. Alves Redol 9, 1000-029 Lisboa, Portugal; 3 Pontifícia Universidade Católica do Rio de Janeiro, Centro Técnico-Científico, Centro de Estudo em Telecomunicações. Rua Marquês de São Vicente 225, Gávea. 22453-900 – Rio de Janeiro, RJ – Brasil; 4 Integrative BioSystems Institute and Dept. Biomedical Engineering, Georgia Institute of Technology and Emory University, 313 Ferst Drive, Atlanta, GA 30332, USA; 5 Dept. Bioinformatics and Computational Biology, University of Texas M.D. Anderson Cancer Center, 1515 Holcombe Blvd, Houston, TX 77030, USA; 6 Faculdade Ciências Médicas – Universidade Nova de Lisboa (FCM-UNL), C Mártires Pátria 130, 1169-056 Lisboa, Portugal

## Abstract

**Background:**

The major difficulty in modeling biological systems from multivariate time series is the identification of parameter sets that endow a model with dynamical behaviors sufficiently similar to the experimental data. Directly related to this parameter estimation issue is the task of identifying the structure and regulation of ill-characterized systems. Both tasks are simplified if the mathematical model is canonical, *i.e*., if it is constructed according to strict guidelines.

**Results:**

In this report, we propose a method for the identification of admissible parameter sets of canonical S-systems from biological time series. The method is based on a Monte Carlo process that is combined with an improved version of our previous parameter optimization algorithm. The method maps the parameter space into the network space, which characterizes the connectivity among components, by creating an ensemble of decoupled S-system models that imitate the dynamical behavior of the time series with sufficient accuracy. The concept of sloppiness is revisited in the context of these S-system models with an exploration not only of different parameter sets that produce similar dynamical behaviors but also different network topologies that yield dynamical similarity.

**Conclusion:**

The proposed parameter estimation methodology was applied to actual time series data from the glycolytic pathway of the bacterium *Lactococcus lactis *and led to ensembles of models with different network topologies. In parallel, the parameter optimization algorithm was applied to the same dynamical data upon imposing a pre-specified network topology derived from prior biological knowledge, and the results from both strategies were compared. The results suggest that the proposed method may serve as a powerful exploration tool for testing hypotheses and the design of new experiments.

## Background

Mathematical models in modern molecular biology have become attractive as compactors of the massive amounts of multidimensional data produced by high-throughput techniques, thus following similar ideas that previously led from reductionism to quantitative inroads into physiology and ecology. In the smaller-dimensional world described by the model structure and its parameters, new experiments are easier to conceive, hypotheses can be tested with greater clarity, and knowledge can be extended with inexpensive computational effort [1].

Generally, mathematical models are implemented with a set of parameters, which give them the flexibility of mapping a range of behaviors into a unifying mathematical framework. Except for singular cases where parameters are directly measured experimentally, parameter estimation from experimental data is an inevitable step in the process of constructing models [2]. A good, first-tier compromise between the need for a closed-form, computable representation of the biological process and the risk of ignoring meaningful parameters of mechanistic, hypothesis-driven reductions can be found in the use of generic, "canonical" modeling frameworks. Specifically, within the framework of biochemical systems theory (BST) [3-6], S-system models (Equation 1) offer a particularly convenient solution because their parameters more or less directly describe the interactions between the components of the system of interest [6].

(1)

In the general S-system form (Equation 1), the time variation of the concentration or amount of each component *X*_*i *_of the system is given by the difference between production and degradation terms. The constant rates *α*_*i *_and *β*_*i *_represent the turnover rates of the production and degradation fluxes and the kinetic rates *g*_*ij *_and *h*_*ij *_quantitatively characterize the influence of the component *X*_*j *_on the production and degradation term of the system component *X*_*i*_, respectively [6,7]. Thus, the network structure and the nature of the interactions driving the phenomenon under investigation are mapped essentially one-to-one onto the parameter values of the model. This modeling framework has been successfully applied to many biochemical systems [6] and can generally be considered a good first default for representing complex biological systems, especially if the governing mechanisms are not well characterized [8]. Automation of the estimation of the high-dimensional parameter set of an S-system model from multivariate time series data has therefore become a widely pursued computational challenge that has been addressed by a wide variety of optimization techniques: from relatively slow global heuristic optimization techniques like genetic algorithms and simulated annealing [9-12] to fast local optimization algorithms such as alternating regression and eigenvector optimization [13,14] among others [15,16]. Most of these optimization algorithms share the strategy of decoupling the differential equation system into a larger, nonlinear algebraic system [6,11,17,18]. This strategy eliminates the need for numerical system integration at each step of the optimization process, which is expensive because S-systems can be numerically stiff, just like most other nonlinear models.

Ironically, the difficulty of finding any numerically integrated S-system model that fits a given set of experimental time series data well is accompanied by the "opposite" problem: many recent publications have pointed out that multiparametric models tend to have the capacity of accommodating whole ranges of parameter values without much affecting the system dynamics [19-28]. Furthermore, it was found that there are typically well-defined directions in the parameter space to which the system dynamics is insensitive, a phenomenon that was termed "sloppiness" [22-25]. Since redundancy appears to be a wide-spread design feature of biological processes, exploring the admissible parameter space of a model and a dataset of interest has relevance that reaches well beyond typical sensitivity analyses of model parameters.

Although the concept of sloppiness has been discussed quite intensely, little attention has been paid to the question of whether or not sloppiness can be translated into the structure of the biological system itself. In other words, is the biological system in reality more or less uniquely parameterized or is there such significant inter-individual variation that we could in principle find large "clouds" of parameter manifestations if we were able to determine the parameters in individual cells or organisms. The answer to this question is not without consequence, because it would affect the definition of what it means to have a good model fit to a given set of data.

In the case of S-systems, the question has further implications. If an admissible parameter cloud, defined by a sufficiently accurate overall fit of some data, permits some kinetic order parameter to be positive, zero, or negative, the interpretation of the estimated model becomes distinctly different. In the first case, the effect is activating, in the second it is negligible, and in the third it is inhibiting. Is such a parameter cloud a computational artifact that would disappear if more data were available, or is it possible that a real biological system would actually allow such different effects of a variable on the system? To reformulate the question, do natural systems only allow for sloppiness in parameter values or also for sloppiness in structure? While the question itself is not new (e.g., [29]), it can presently not be answered in generality and with reliability.

In this report, we address the intrinsic redundancies in the interactions between biological system components by proposing an embedding method for S-system parameter space estimation based on a Monte Carlo process that is combined with an improved version of our previous parameter optimization algorithm. We apply this methodology to experimental time series data characterizing the glycolytic pathway in the bacterium *Lactococcus lactis *[30-32]. In the same context we explore the concept of sloppiness in S-systems by studying the implications of admissible ensembles of models that dynamically represent the data well but lead to different interpretations.

### Neutral solution analysis

This section describes the concepts of the proposed analysis of neutral solution spaces, *i.e*., of multiple model parameter sets with similar dynamics; all technical details are presented in the later *Methods *section. To characterize the neutral solution spaces, we propose a Monte Carlo (MC) random walk process [33], which is sped up by a nonlinear optimization algorithm that allows us to assess S-system parameter sets (forming the "neutral space") that give the system a similar dynamical behavior as it had been measured experimentally in the form of time series data. Differently from similar methodologies suggested in the literature [22,23,25,26], the proposed approach is performed with the decoupled form of the system [34], which allows analysis of one system equation at a time. In this fashion, problems with numerical integration of differential equations, which is otherwise needed at each step of the MC process, are avoided. Thus, we suppose in the following that the time series of all components are available and have been smoothed, thus permitting the numerical estimation of their first derivatives at each point of the time series. For each system component, a series of steps is performed as follows.

First, using the smoothed time series of all components *X*_*i*_, as well as their numerically estimated derivatives, the system of differential equations is converted into a system of algebraic equations (Equation 2) [11,18,34]. Second, an optimization algorithm is applied to this algebraic system, leading to an optimal parameter set that matches the algebraic equations with the observed systems dynamics. This optimal parameter set is the starting point of the MC process. A cost function *C *is defined to quantify changes in behavior of the decoupled system resulting from perturbations in the parameter set (Equation 16). The Hessian matrix of *C *is calculated at the optimal parameter set and used to guide subsequent, artificial parameter perturbations, which collectively form the MC random walk [22,25]. At each MC step, the parameter set is perturbed (using the eigenvectors of the Hessian matrix [35]) and then used as initial guess for the optimization algorithm, however, with an earlier stop criterion. This premature termination prevents the algorithm from converging to the same local optimal point and is accompanied by a (small) residual error. The cost function *C *is evaluated with the new local parameter set, and this parameter set is accepted for the next iteration with a certain probability (Equation 18). Any parameter sets satisfying the conditions of a predefined behavioral class (*e.g*., with a cost function value smaller than a threshold) are recorded. At the conclusion of the MC process, the recorded collection of parameter sets contains solutions of the decoupled system that adhere to the specified behavioral class.

After the MC process has been run for each system component, instances from the collections of parameter sets for all variables are randomly sampled to recouple the models by means of numerical integration. Although the decoupled, algebraic form offers the advantage of avoiding numerical integration problems, it is not guaranteed that the recoupled system will lead to an accurate, comprehensive solution. The *Methods *and *Results *sections provide detailed descriptions of the proposed techniques and outcomes.

## Results

After preliminary, successful tests with simulated data (see supplementary material), we applied the proposed optimization algorithm to actual time series. These data consist of metabolite profiles from the glycolytic pathway of the bacterium *Lactococcus lactis*, which were obtained with *in vivo *NMR experiments [30,31]. For modeling purposes, the concentrations of the metabolites were coded as follows: glucose – *X*_1_; glucose 6-phosphate (G6P) – *X*_2_; fructose 1, 6-bisphosphate (FBP) – *X*_3_; phosphoenolpyruvate (PEP) – *X*_4_; lactate – *X*_5_; acetate – *X*_6_. As a pre-processing step for the parameter optimization, the time series were smoothed/denoised and their slopes (numerical first derivatives) were estimated as shown in previous work on non-stationary noise filtering [36].

**Figure 1:**
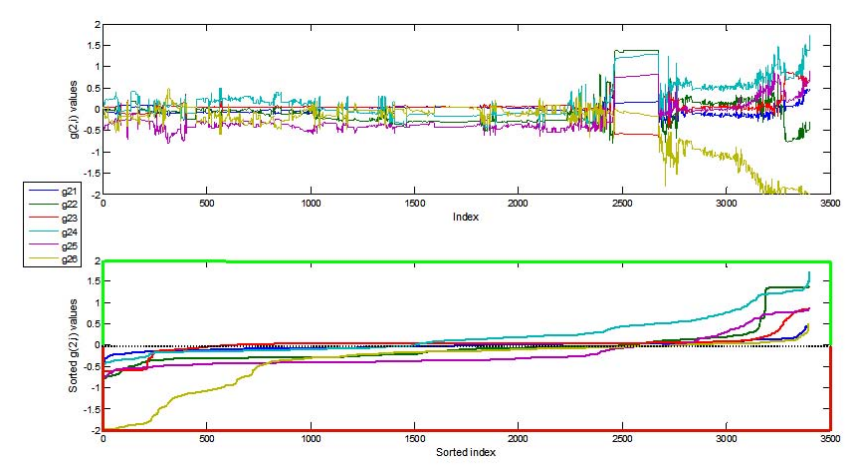
**Kinetic orders estimated for the G6P (*X*_2_) production term with potential inclusion of all system variables**. A) The two indices refer to the two species considered by the interaction; for instance, g_26 _indicates the effect of *X*_6 _(acetate) on G6P production. B) The same parameter sets as shown in A, but ordered individually by magnitude, showing the possible variation admissible each parameter. The light green y-axis represents the region of parameter space with possible activation interaction; analogously, the light red y-axis represents the region of possible inhibition interaction.

Initially, the proposed algorithm exclusively used the time series of all metabolites in the parameter optimization, representing the case where no knowledge about the network connectivity is at hand. The optimal parameter set of each metabolite was separately translated into the eigenspace of the solution and subsequently fed into the MC process. The parameters were then perturbed and an ensemble of models was created as described in the *Methods *section. The parameter sets were selected based on a behavioral class [37], which was defined by a residual less than 5 (see Equation 15). As an example, Figure 1 shows all parameter sets for the G6P production term that fall within the defined behavioral class. After ordering, these parameters have the same distribution pattern (Figure 1B). Similar results were found for all other metabolites.

As an alternative, we performed a MC random walk in the original parameter space, as opposed to the eigenspace. Interestingly, although not surprising in the end, the results did not present as wide a range as was found in the exploration of the parameter set in the eigenspace. The difference in outcomes is explained by the compensation of errors between production and degradation terms: perturbations in the eigenspace of the matrix *W *affect both production and degradation terms while perturbing only one parameter does not always maintain the balance between the two terms. In other words, given one of the S-system's terms (production or degradation) and the vector of slopes, the complementary term can be obtained by multiple linear regression, which has been shown not to be sloppy [24]).

**Figure 2:**
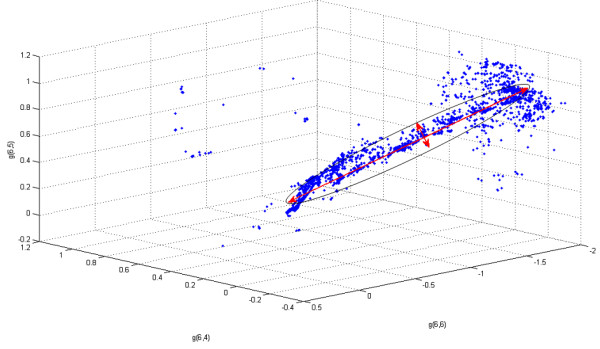
**Quantification of sloppiness for one of the equations of the *Lactococcus *model**. The figure shows the 3-D projection of the ellipsoid that represents the region of the parameter space that produces similar dynamical behaviors. The arrows show the direction of the sloppy and stiff directions in the 3-D projection, corresponding respectively to eigenvectors with small and large eigenvalues of the Hessian matrix of the cost function.

As expected, eigenvalues of the Hessian matrix fall within a sparse range [22-25,38], thereby elucidating the stiff and sloppy directions (see *Additional file*1). The region of the parameter space that produces similar dynamical model behaviors can be approximated as an ellipsoid whose main axes are given by the direction of the eigenvectors of the Hessian matrix (see Figure 2) and whose width is inversely proportional to the squared root of the corresponding eigenvalue [22-25,38]. A projection of the ellipsoid into three-dimensional space for the acetate production parameters g_64_, g_65 _and g_66 _is shown in Figure 2. Revisiting reasons discussed for other modeling frameworks [22-25,27], sloppiness can be explained in the proposed approach by the neutral space of solutions for Equation 6 and consequently Equation 8. Specifically, given the "right" linear combination of the eigenspace of the matrix *W*, any vector resulting from stretching or shrinking this combination is also a solution of Equation 6, however with different parameter values.

**Figure 3:**
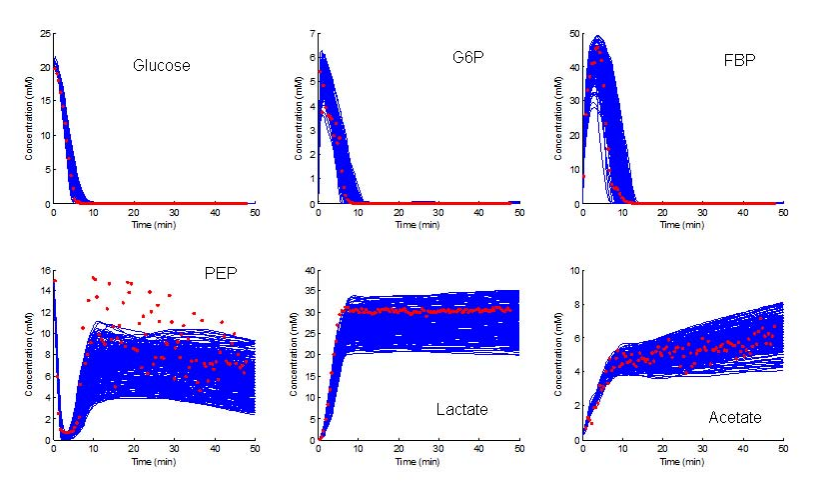
**Ensembles of 500 models generated by randomly sampling parameter sets of all metabolites from the outcomes of metabolite specific MC random walk processes**.

Ideally, all combinations of parameter sets found for the individual metabolites would generate recoupled models that fit the data upon numerical integration, within some error bound. Given the large number of parameters (at the order of 10^14 ^combinations), a full exploration of this statement is not possible. In order to ameliorate the expensive combinatorial issue of assessing the trajectories of the recoupled systems, the parameter set for each metabolite generated from the MC process was randomly selected and the resulting system numerically integrated. This process was repeated 500 times and the results are presented in Figure 3.

**Figure 4:**
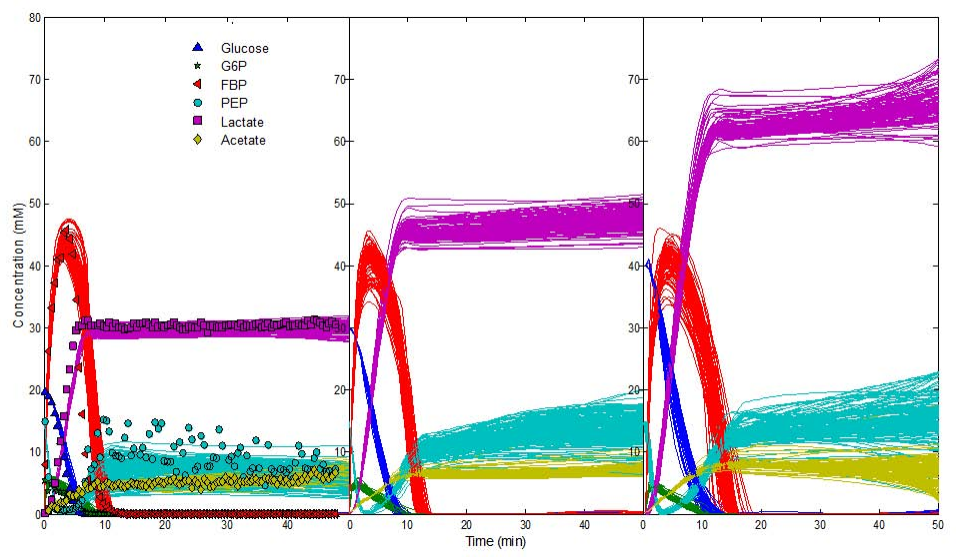
**New ensemble of models**. The systems were integrated with different initial concentrations for glucose substrate, namely with 20, 30 and 40 mM; results are shown, respectively, in panels a, b and c.

As can be seen from Figure 3, the uncertainties and bounds in the prediction for most metabolites are actually close to the observed data. A notable exception is lactate, where the measured data contain little noise. Because PEP is the main precursor of lactate in the model [31] (pyruvate is not explicitly modeled), the class of predictions of the PEP dynamics was re-sampled for residuals less than 1. Furthermore, the newly sampled systems were integrated with glucose supplied in three different initial concentrations, namely 20, 30 and 40 mM. The results of these predictions are shown in Figure 4. For all three different scenarios the new ensemble model predictions provide accurate descriptions of the observed dynamics for the concentrations of lactate and the other metabolites in the model, except for the noticeable undershooting of PEP (see [30,31] for information on the responses of the system to different initial glucose concentrations).

Although the models found by the proposed procedure accurately describe the dynamics observed in the experimental data, none of the parameter sets match well with the network topologies found in the literature, suggesting that distinctly different parameter combinations, and not just sloppy versions of some parameter set, area able to match these data. A variety of techniques can be applied to the ensemble in order to compare different models [37,39], to cluster models by means of transformation groups, or even reduce them to a smaller subset [20]. One possible avenue for further analysis of this vast parameter space is by creating groups defined by different behavior classes based on biological and dynamical information [37,39].

**Figure 5:**
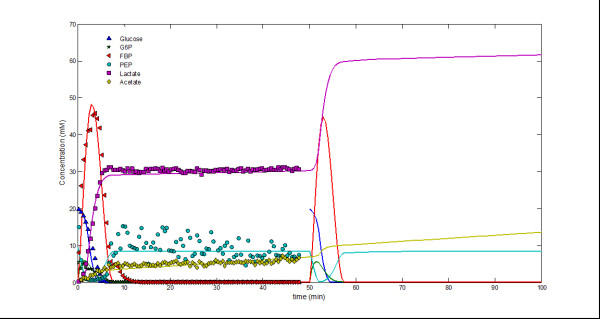
**Glucose double pulse simulation**. A second 20 mM glucose pulse was supplied to the system after 50 min, resulting in the further accumulation of lactate and acetate.

Regardless of specific follow-up analyses, one of the purposes of this work is to demonstrate that the proposed optimization algorithm can be effectively applied to the parameter identification of specific networks, for instance, by taking kinetic parameters of some component *X*_*j *_out of the optimization process of *X*_*i*_. Conversely, previous knowledge can be used to restrict the values of the *g *and *h *interaction parameters. For example, existing knowledge about *Lactococcus lactis *primary metabolism [30-32,40] provides precise clues about which interactions are reasonable. To analyze the benefit of information outside the time series data, we applied the proposed optimization algorithm to the same *Lactococcus lactis *data, this time however constraining some parameter values with the pre-existing information. The simulation results with the resulting system (Figure 5; see equations in *Additional file*1) not only describe the data but also agree with the double pulse of glucose described in [32].

Exactly as described before, the system resulting from this combined approach could be used as the starting point of a MC random walk process, and the same analysis of sloppiness and behavior classes could be performed. In order to assess the accuracy of the solutions for large systems, the optimization algorithm was also tested with a symbolic genetic network model consisting of 30 components [41] (see *Additional file*1). Because the algorithm performs the parameter optimization with the decoupled form of the system, its complexity is linear with the number of the system's components [14]. Thus, rather than system dimension, the real limitation of the optimization algorithm is the time series dynamics. Poor dynamical variability (components close to the steady-state) and collinear time series will result in a conditioning deficiency of the matrix *L*, causing numerical problems with the inverse operations and misleading the convergence. Despite the successful retrieval of the 30-dimensional system dynamics, the algorithm's limitation becomes more evident with large systems (higher possibility of having time series portions with collinear components, resulting in ill-conditioned blocks in the matrix *L *[42]). This drawback is partially resolved with the regularization technique presented in the *Methods *section. Also, problems of this nature can be prevented by removing the collinear components from the matrix *L *or placing them as independent variables (variables that do not take place on the parameter optimization [6]). Of course, this issue is drastically diminished when a chosen network topology constraints the matrix *L*.

## Discussion and Conclusion

Within the new field of systems biology, an extraordinary effort has been devoted to kinetic modeling with the aim of understanding biological processes better [1,43,44]. A very significant part of this effort has been directly related to the identification of model parameters [2,45-47]. Until recently, the quality of the estimated parameter values was judged by the fit of experimental data. However, new analyses have pointed out the importance of other criteria, such as extrapolability and error compensation among terms [48], which is in some sense related to the sloppiness of admissible parameter sets and the fact that different parameter values can generate similar dynamical behaviors in nonlinear biological systems models [22,23,25-27]. These observations have a direct impact on the robustness of models, which may translate into robustness of the biological system itself, which may become apparent in tolerance to mutations, changes in gene expression, and insensitivity to modest changes in environmental conditions. This need for physiological robustness implies that biochemical networks should be able to preserve their dynamical properties within reasonable ranges of their kinetic parameters [49].

In this report, we present an extension of our previous optimization algorithm for S-system parameter identification from time series data [14]. The proposed method turned out to be faster and more accurate than its predecessor (see *Additional file*1) and was used here in combination with a Monte Carlo random walk technique to explore the space of admissible parameter sets of S-system models. This strategy allowed us to explore the concept of sloppy models. The results indicate that both, a fully integrated and a decoupled model, can be sloppy. We also reanalyzed time series of the concentrations of six metabolites within the glycolytic pathway of the bacterium *Lactococcus lactis *and demonstrated how a sloppiness analysis can elucidate the admissible parameter space and ultimately lead to more reliable estimates.

The central result reported here is that a diversity of parameter sets may produce quasi-isomorphic dynamics for S-system models. Most of the parameter variations extended to both positive and negative parts of the parameter space (Figure 1B). This result is interesting, because it could be the consequence of two distinctly different scenarios. First, it could reflect redundancy or sloppiness caused by insufficient data. In other words, the data are not informative enough to distinguish between alternative models that fit equally well. In the past, such situations have often been "resolved" by setting as many parameters to zero as possible with the set of admissible solutions, borrowing arguments of parsimony or Ockham's razor. The important feature of this scenario is that further experimental data, maybe obtained under similar yet sufficiently different conditions, would declare one of the candidate models the (sole) winner. The second possible root cause of distinct, well-fitting parameter sets is the actual natural co-existence of different regulatory signals between components (inhibition *versus *activation) or different regulatory networks (some with and some without particular regulatory signals). Thus, otherwise similar cells or organisms would function under slightly different regulatory regimens. The difference between these scenarios is conceptually similar to the distinction between uncertainty and variability, which was discussed intensely in the 1980s and 1990s within the fields of risk assessment and exposure analysis [50]. The former case of uncertainty (due to insufficient data) is only valid if the model is, in principle, uniquely identifiable and structurally distinguishable [19,51] from among all feasible S-systems. This aspect raises the possibility that a sloppy model could be unidentifiable and that the sloppy directions, which are given by the possible parameter combinations [22] could be a measure of non-identifiability [51]. For S-systems this argumentation can be extended. Our results show that an ensemble of S-systems could be interpreted as a collection of structurally indistinguishable and unidentifiable models [19]. This conjecture would explain the range of variability (negative, zero, positive) of the parameter sets that were found. Furthermore, the identifiability characteristic of the mathematical framework could be associated with the robustness of the biological system to environmental changes. Analogously, the structure distinguishability characteristic could be, in some sense, associated with the robustness of the biological system to mutations that change the network of interaction among components. A more rigorous study of S-system identifiability and distinguishability will be needed to reveal more concrete conclusions and mathematical implications [52,53].

Whether the distinction between parameter sloppiness and structural distinguishability in biological models is an important issue will have to await further investigation. Nevertheless, it is clear that extrapolability in the generic network identification problem from time series data is a more complex task than the computational fitting of a model. One should expect that this observation is true for any model structure, but it was shown here that S-system models allow its exploration in a most translucent fashion.

The assessment of sloppiness provides valuable information that can be extracted from the prediction of the ensemble of models and through the investigation of behavioral classes that differ in their dynamical features (*e.g*. overshoots or response time). These classes may be further reduced by biological knowledge such as biochemical, physico-chemical, or thermodynamic constraints [54,55]. The proposed techniques could also serve as a powerful exploration tool for the testing of hypotheses and the design of new experiments. Moreover, the union of the proposed optimization algorithm with statistical methods could also result in a robust network inference implementation [56].

Maybe the most important value of this report is the clear definition of a framework to explore sloppiness, robustness and evolutionary innovation [38,49,57], where neutral parameter spaces (system with essentially the same dynamical behavior) are merged with neutral networks [49,57] into one unique structure. This approach could reveal insights into how different metabolic networks could possibly have been changed during the evolutionary process.

## Methods

The method described here is an extension of our previous work in S-systems parameter estimation [14]. Exactly as its predecessor, the proposed algorithm exploits the advantages of the numerical decoupling of S-systems, which allows that each component has its own optimization process [6,11]. However, the optimization is now performed in a transformed equation of the decoupled form. This transformation enhances the ability of the algorithm to reach local optimal points. Given a time series collection *X*_*m*_(*t*_*i*_) of the concentrations of species or metabolites *m *= [1,..., *M*] in the time interval *t*_*i *_= [*t*_1_,..., *t*_*N*_*] *and their respective slope values *S*_*m*_(*t*_*i*_) estimated in the same time interval, one can numerically decouple the S-system equations and write a semi-linearization (only the left-hand side is linearized) form for each species *m *as follows

(2)

where

(3)

and *Vp*_*m *_= [log *α*_*m *_*g*_*m*1 _*g*_*m*2 _⋯ *g*_*mM*_] is the parameter vector for the production term. In Equation (2), the time point index *t*_*i *_is suppressed in order to keep a simple notation. If a multiple regression step is applied to the system in Equation (2), the production parameter vector can be written as

(4)

The matrix *L*^+ ^is the Moore-Penrose inverse of the matrix *L*, given by *L*^+ ^= (*L*^*T*^L)^-1^*L*^*T*^. Substituting Equation (4) into Equation (2), we obtain the following eigenvector problem [14,58]:

(5)

In this equation, the matrix *W *= *LL*^+ ^is idempotent with *M*+1 unit eigenvalues, while the remaining eigenvalues are zero. It is clear from Equation (5) that not only  but also  are eigenvectors of *W *corresponding to eigenvalue 1. If Equations (2), (4) and (5) are rewritten in order to isolate the degradation parameter vector instead of the production parameter vector, the vector  also belongs to the eigenspace. Standard routines for eigenvalue analysis can easily calculate the eigenvectors of matrix *W*. However, this does not imply that any of these eigenvectors will map onto the "correct" parameter set. In fact, these vectors form the eigenspace of *W *corresponding to eigenvalue 1, leading to the conclusion that the true parameter set can be formulated as a linear combination of these eigenvectors. Thus, let *EigS *be a matrix where the columns are the eigenvectors with correspondent eigenvalues 1. Then the following vectors can be defined:

(6)

or

(7)

In Equations (6) and (7), *ψ *and *δ *are (*M+*1)-dimensional vectors that represent an arbitrary linear combination of the eigenvectors of *W*. We can use Equation (7) to write the decoupled form of the S-system as

(8)

Equation (8), once written as a function of the production and degradation parameter vectors, can now be seen as a function of the vectors *ψ *and *δ*. Using the uniqueness of the mapping between the vectors *ψ *and *δ*,

(9)

we can rewrite Equaiton (8) as a function of only the vector *δ*. For the estimation of vector *δ *and consequently of vector *ψ*, we define a cost function as the sum of squared residuals between the two sides of Eq. (8).

(10)

where  is the right-hand side of the Equation (8). Thus, Equation (10) leads to the following gradient equations

(11)

or

(12)

where

(13)

The symbols and represent the Hadamard and Shanghai Jiao Tong (SJT) product respectively [42,59], defined as

(14)

In Equations (11) and (12), *υ *is the argument of the logarithm in Equation (10). We use the Levenberg-Marquardt routine [60] for the minimization of *F *with the following nonlinear constraints

(14a)

In these inequality constraints (see also Equation (7)), the pairs *lcb-ucb *and *lkb-ukb *are the intervals (lower and upper bounds) for the rate constants and kinetic orders respectively. Preliminary tests with a simulated systems show the potential and accuracy of the algorithm (see *Additional file*1). Without change in the gradient equations, a Tikhonov regularization [61] was also implemented in order to overcome potential problems with the ill-conditioning of the matrix *L*, resulting in the following matrix:

(15)

where *λ *is the regularization parameter and *I *the identity matrix.

### Sloppiness in S-systems models

Sloppiness has been proposed as a nearly universal characteristic of parameter sensitivity from multiparametric nonlinear models (see definition of 'universal' in [22]). In a nutshell, a model is sloppy if it is markedly more sensitive to some parameter combinations than to others. This feature has been shown to be the rule rather than the exception among biological systems models [22]. One characteristic attribute of sloppiness is a diversity of parameter sets that produce similar dynamical behaviors. If a cost function is defined to quantify the variation in dynamical behavior one can visualize sloppiness in terms of the eigenvalues of the Hessian of this cost function. These eigenvalues are typically very sparse within a large range, suggesting that the model is more sensitivity to certain parameter combinations (eigenvectors with largest eigenvalues – stiff combinations) than to others (eigenvectors with smallest eigenvalues – sloppy combinations) [22-25].

For S-system models, the diversity in the parameter space is to be interpreted not only as a variation in the kinetic constants but also as redundancy in the topology of the biological network or as alternative topologies associated with the same phenotype. Either interpretation has no effect on the usefulness of the model from the predictability point of view as we discuss in the *Results *section. Indeed, it has been shown that sloppy models can lead to accurate predictions (*e.g*., [24,38]).

### Decoupled ensembles of S-system models

Recently, a Monte Carlo random walk method [33] in the parameter space of multiparametric system biology models was suggested in order to assess uncertainties in model parameters and model structure [22,23,25,26,38]. In this context, S-system models are particularly convenient because they map any network topology directly onto its parameter values (*g *and *h*) with no change in model structure [6]. Therefore, we propose a walk in the parameter space of the S-system and proffer that it corresponds to a walk in the 'topology space'. This idea is explored here by combining of the Monte Carlo approach with a nonlinear optimization algorithm. In contrast to relevant work in the literature, but in accordance with our optimization method, the random walk is performed separately with the decoupled S-system format for each system component. This strategy avoids difficulties encountered in the numerical integration of stiff equations and poses no significant loss of information, under the reasonable assumption that there are no bifurcation points within the considered parameter ranges. Thus, we define the following cost function, which quantifies variations in the first derivatives of a variable with respect to variations in the parameter set Δ*δ*:

(16)

Here, δ* is the optimal parameter set found using the proposed optimization algorithm. Evaluation of the Hessian matrix,

(17)

allows us to analyze the sensitivity of the first derivative of each component of the system in relation to its parameters. This Hessian is also used to guide the Monte Carlo random walk, which selects the next perturbations within the parameter set. Thus, the Monte Carlo random walk starts with the optimal vector δ* as initial condition, randomly selects one component of this vector and perturbs it proportional to the Hessian eigenvector (see [25,35]), and uses the new vector as initial condition for the proposed optimization algorithm, which now has an early stop criterion (number of iterations). The new optimal vector Δ*δ *(*i.e*., the outcome of the optimization algorithm) is accepted as the next step of the process with probability

(18)

[26], where

(19)

is the probability distribution of the parameter set *δ *given the model for the slopes *δ m*(δ*). In Equation (19), *k *is a normalization factor (that vanishes in the probability of acceptance [26], Eq. (18)) and *σ *is the standard deviation of the slopes *S*_*m*_. If a new vector *δ *is accepted for the next step, the hessian matrix is recalculated and the process follows as described above. This strategy permits a more detailed exploration of the parameter space where the MC steps are taken in the sloppy directions. A small range for the number of iterations of the optimization algorithm (early stop criteria) was tested and no significant differences were observed. Relative large numbers were avoided in order to prevent the algorithm convergence to the same neighborhood. After the optimization process, the vectors *δ *are mapped onto the parameter degradation vector *Vd*_*m *_= [log *β*_*m *_*h*_*m*1_*h*_*m*2 _⋯ *h*_*mM*_]^*T *^and consequently onto the parameter production term *Vp*_*m *_= [log *α*_*m *_*g*_*m*1 _*g*_*m*2 _⋯ *g*_*mM*_]^*T*^, restating the decoupled system in its original parameters. All the numerical integrations presented in this report were carried out by the MATLAB^® ^ode23s routine. The rate constants were optimized within the range [0.1, 300] while the kinetic order were optimized within the range [-2, 2]. All the results shown in this report can be reproduced with the freely available MATLAB^® ^scripts (see *Additional file*2).

## Competing interests

The authors declare that they have no competing interests.

## Authors' contributions

MV and MAGMM conceived the core of the optimization method. SV participated in the analysis and systematization of the statistical method used in this work. EOV initiated the field of network identification with S-systems and supervised activities leading to this paper. JSA conceived the ideas of automating the identification of S-systems and creating a model pipeline. All authors contributed to in the preparation of the manuscript.

## Supplementary Material

Additional file 1 **Numerical tests.** This additional file provide further numerical tests using the proposed methodologies. Click here for file

Additional file 2 **Optimization algorithm implementation.** This additional file provide the Matlab scripts of the optimization algorithm proposed in the main text. Click here for file

## References

[B1] Westerhoff HV, Kolodkin A, Conradie R, Wilkinson SJ, Bruggeman FJ, Krab K, van Schuppen JH, Hardin H, Bakker BM, Mone MJ (2009). Systems biology towards life in silico: mathematics of the control of living cells. J Math Biol.

[B2] Banga JR (2008). Optimization in computational systems biology. BMC Syst Biol.

[B3] Savageau MA (1969). Biochemical systems analysis. I. Some mathematical properties of the rate law for the component enzymatic reactions. J Theor Biol.

[B4] Savageau MA (1969). Biochemical systems analysis. II. The steady-state solutions for an n-pool system using a power-law approximation. J Theor Biol.

[B5] Savageau MA (1970). Biochemical systems analysis. 3. Dynamic solutions using a power-law approximation. J Theor Biol.

[B6] Voit EO (2000). Computational analysis of biochemical systems: a practical guide for biochemists and molecular biologists.

[B7] Savageau MA (1976). Biochemical systems analysis: a study of function and design in molecular biology.

[B8] Hecker M, Lambeck S, Toepfer S, van Someren E, Guthke R (2009). Gene regulatory network inference: data integration in dynamic models-a review. Biosystems.

[B9] Kikuchi S, Tominaga D, Arita M, Takahashi K, Tomita M (2003). Dynamic modeling of genetic networks using genetic algorithm and S-system. Bioinformatics.

[B10] Gonzalez OR, Kuper C, Jung K, Naval PC, Mendoza E (2007). Parameter estimation using Simulated Annealing for S-system models of biochemical networks. Bioinformatics.

[B11] Voit EO, Almeida J (2004). Decoupling dynamical systems for pathway identification from metabolic profiles. Bioinformatics.

[B12] Liu PK, Wang FS (2008). Inference of biochemical network models in S-system using multiobjective optimization approach. Bioinformatics.

[B13] Chou IC, Martens H, Voit EO (2006). Parameter estimation in biochemical systems models with alternating regression. Theor Biol Med Model.

[B14] Vilela M, Chou IC, Vinga S, Vasconcelos AT, Voit EO, Almeida JS (2008). Parameter optimization in S-system models. BMC Syst Biol.

[B15] Sorribas A, Cascante M (1994). Structure identifiability in metabolic pathways: parameter estimation in models based on the power-law formalism. Biochem J.

[B16] Sorribas A, Samitier S, Canela EI, Cascante M (1993). Metabolic pathway characterization from transient response data obtained in situ: parameter estimation in S-system models. J Theor Biol.

[B17] Voit EO, Savageau MA (1982). Power-law approach to modeling biological systems; III. Methods of analysis. J Ferment Technol.

[B18] Almeida JS, Voit EO (2003). Neural-network-based parameter estimation in S-system models of biological networks. Genome Inform.

[B19] Walter E, Pronzato L (1996). On the identifiability and distinguishability of nonlinear parametric models. Mathematics and Computers in Simulation.

[B20] Voit EO (1992). Symmetries of S-systems. Math Biosci.

[B21] Sands PJ, Voit EO (1996). Flux-based estimation of parameters in S-systems. Ecol Modeling.

[B22] Gutenkunst RN, Waterfall JJ, Casey FP, Brown KS, Myers CR, Sethna JP (2007). Universally sloppy parameter sensitivities in systems biology models. PLoS Comput Biol.

[B23] Gutenkunst RN, Casey FP, Waterfall JJ, Myers CR, Sethna JP (2007). Extracting falsifiable predictions from sloppy models. Ann N Y Acad Sci.

[B24] Waterfall JJ, Casey FP, Gutenkunst RN, Brown KS, Myers CR, Brouwer PW, Elser V, Sethna JP (2006). Sloppy-model universality class and the Vandermonde matrix. Phys Rev Lett.

[B25] Brown KS, Sethna JP (2003). Statistical mechanical approaches to models with many poorly known parameters. Phys Rev E Stat Nonlin Soft Matter Phys.

[B26] Battogtokh D, Asch DK, Case ME, Arnold J, Schuttler HB (2002). An ensemble method for identifying regulatory circuits with special reference to the qa gene cluster of Neurospora crassa. Proc Natl Acad Sci USA.

[B27] Piazza M, Feng XJ, Rabinowitz JD, Rabitz H (2008). Diverse metabolic model parameters generate similar methionine cycle dynamics. J Theor Biol.

[B28] Barbano PE, Spivak M, Flajolet M, Nairn AC, Greengard P, Greengard L (2007). A mathematical tool for exploring the dynamics of biological networks. Proc Natl Acad Sci USA.

[B29] Voit EO, Savageau MA (1982). Power-law approach to modeling biological systems; II. Application to ethanol production. J Ferment Technol.

[B30] Ramos A, Neves AR, Santos H (2002). Metabolism of lactic acid bacteria studied by nuclear magnetic resonance. Antonie Van Leeuwenhoek.

[B31] Neves AR, Pool WA, Kok J, Kuipers OP, Santos H (2005). Overview on sugar metabolism and its control in Lactococcus lactis – the input from in vivo NMR. FEMS Microbiol Rev.

[B32] Voit E, Neves AR, Santos H (2006). The intricate side of systems biology. Proc Natl Acad Sci USA.

[B33] Metropolis N, Ulam S (1949). The Monte Carlo Method. J Amer Stat Assoc.

[B34] Voit EO, Ferreira AEN (2000). Computational analysis of biochemical systems: a practical guide for biochemists and molecular biologists.

[B35] Brown KS, Hill CC, Calero GA, Myers CR, Lee KH, Sethna JP, Cerione RA (2004). The statistical mechanics of complex signaling networks: nerve growth factor signaling. Phys Biol.

[B36] Vilela M, Borges CC, Vinga S, Vasconcelos AT, Santos H, Voit EO, Almeida JS (2007). Automated smoother for the numerical decoupling of dynamics models. BMC Bioinformatics.

[B37] Alves R, Savageau MA (2000). Systemic properties of ensembles of metabolic networks: application of graphical and statistical methods to simple unbranched pathways. Bioinformatics.

[B38] Daniels BC, Chen YJ, Sethna JP, Gutenkunst RN, Myers CR (2008). Sloppiness, robustness, and evolvability in systems biology. Curr Opin Biotechnol.

[B39] Alves R, Savageau MA (2000). Comparing systemic properties of ensembles of biological networks by graphical and statistical methods. Bioinformatics.

[B40] Voit EO, Almeida J, Marino S, Lall R, Goel G, Neves AR, Santos H (2006). Regulation of glycolysis in Lactococcus lactis: an unfinished systems biological case study. Syst Biol (Stevenage).

[B41] Kimura S, Ide K, Kashihara A, Kano M, Hatakeyama M, Masui R, Nakagawa N, Yokoyama S, Kuramitsu S, Konagaya A (2005). Inference of S-system models of genetic networks using a cooperative coevolutionary algorithm. Bioinformatics.

[B42] Magnus JR, Neudecker H (1988). Matrix differential calculus with applications in statistics and econometrics.

[B43] Fisher J, Henzinger TA (2007). Executable cell biology. Nat Biotechnol.

[B44] Palsson B (2006). Systems biology: properties of reconstructed networks.

[B45] Peifer M, Timmer J (2007). Parameter estimation in ordinary differential equations for biochemical processes using the method of multiple shooting. IET Syst Biol.

[B46] Balsa-Canto E, Peifer M, Banga JR, Timmer J, Fleck C (2008). Hybrid optimization method with general switching strategy for parameter estimation. BMC Syst Biol.

[B47] Kitayama T, Kinoshita A, Sugimoto M, Nakayama Y, Tomita M (2006). A simplified method for power-law modelling of metabolic pathways from time-course data and steady-state flux profiles. Theor Biol Med Model.

[B48] Goel G, Chou IC, Voit EO (2008). System estimation from metabolic time-series data. Bioinformatics.

[B49] Ciliberti S, Martin OC, Wagner A (2007). Robustness can evolve gradually in complex regulatory gene networks with varying topology. PLoS Comput Biol.

[B50] Cullen AC, Frey HC (1999). Probabilistic techniques in exposure assessment: a handbook for dealing with variability and uncertainty in models and inputs.

[B51] Cobelli C, Distefano JJ (1980). Parameter and Structural Identifiability Concepts and Ambiguities – a Critical-Review and Analysis. American Journal of Physiology.

[B52] Ljung L, Glad T (1994). On Global Identifiability for Arbitrary Model Parametrizations. Automatica.

[B53] Vinga S, Thomaseth K, Lemos JM, Neves AR, H S, AT F (2008). Structural analysis of metabolic networks: a case study on Lactococcus lactis. 8th Portuguese Conference on Automatic Control: 2008; Vila Real, Portugal.

[B54] Liebermeister W, Klipp E (2006). Bringing metabolic networks to life: convenience rate law and thermodynamic constraints. Theor Biol Med Model.

[B55] Ederer M, Gilles ED (2007). Thermodynamically feasible kinetic models of reaction networks. Biophys J.

[B56] Price ND, Shmulevich I (2007). Biochemical and statistical network models for systems biology. Curr Opin Biotechnol.

[B57] Ciliberti S, Martin OC, Wagner A (2007). Innovation and robustness in complex regulatory gene networks. Proc Natl Acad Sci USA.

[B58] Lay DC (2006). Linear algebra and it's applications.

[B59] W Chen CS, He W (2000). The DQ solution of geometrically nonlinear bending of orthotropic rectangular plates by using Hadamard and SJT product. Computers & Structures.

[B60] Marquardt DW (1963). An algorithm for least-squares of nonlinear parameters. SIAM J Appl Math.

[B61] Tikhonov A (1943). On the stability of inverse problems. Dokl Akad Nauk SSSR.

