# Supplementary material for: Identification of neutral biochemical network models from time series data
Source: BMC Syst Biol. 2009 May 5;3:47. doi: 10.1186/1752-0509-3-47 (PMC2694766; doi:10.1186/1752-0509-3-47)
Supplement: Additional file 1 — Numerical tests. This additional file provide further numerical tests using the proposed methodologies. [file 1752-0509-3-47-S1.doc]

Supplementary material to

***Identification of Neutral Sets of Biochemical Systems Models from Time Series Data***

Marco Vilela,Susana Vinga, Marco Antonio Grivet Mattoso Maia, Eberhard O. Voit, Jonas S. Almeida.

In order to assess the accuracy of the proposed parameter optimization algorithm, we performed a set of tests using the following systems found in the literature [1-3]

, (1)

, (2)

and

(3)

For all the above systems (equations 1, 2 and 3), the proposed optimization algorithm found the original parameter set with an average time of 7 sec per variable (X_i_). The derivative of each variable was calculated directly from the system’s equations and variables were used for the optimization of all parameter sets, corresponding to the case where no information about the network topology is known. A similar convergence pattern to the one shown in figure 1 was found for all parameter sets of the 3 systems. These experiments can be reproduced with the provided MATLAB® scripts.


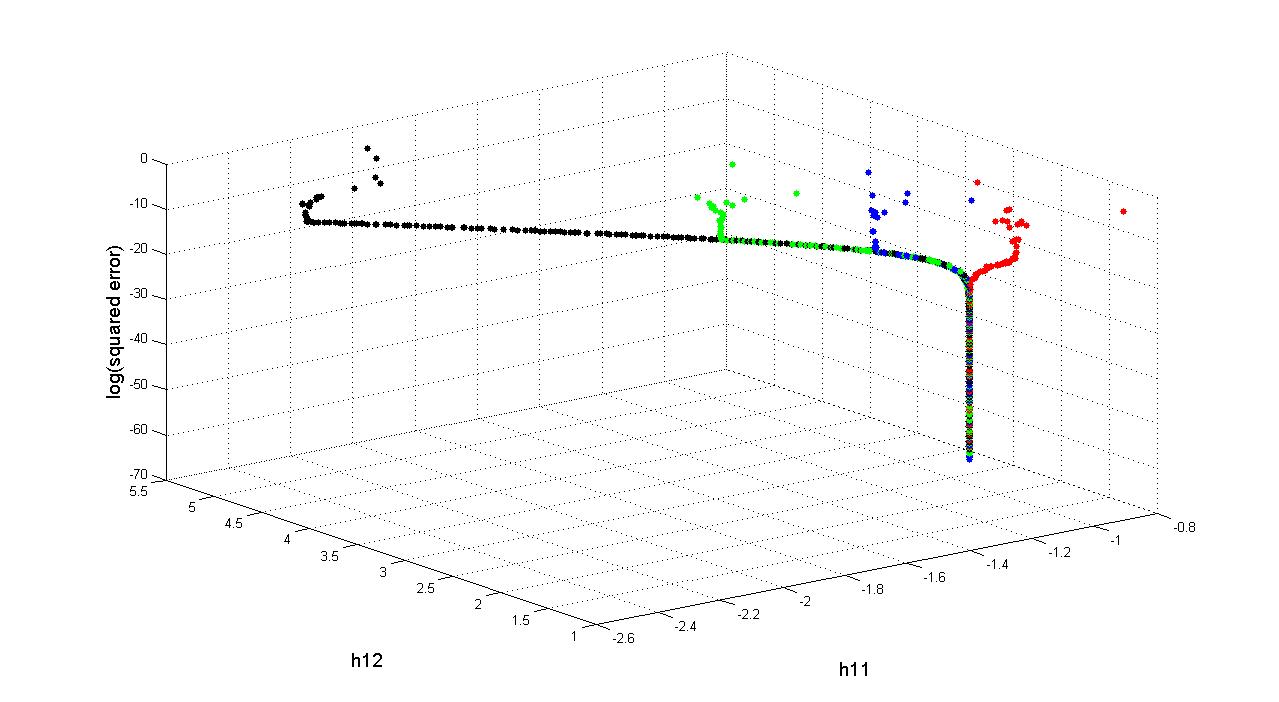


Figure 1 – Convergence pattern. Path of the kinetic parameters h_11_ and h_12_ of the 4-dimensional system during the optimization process. Different colors show different initial conditions, spaced all over the feasible range.

30-Dimensional system

In order to assess the accuracy and limitation of our algorithm, we performed the parameter optimization on a 30-dimensional system that represents a genetic network [3]. To avoid time windows with collinear components, we changed some kinetic orders and initial concentrations from those ones published in [3] during the data generation.


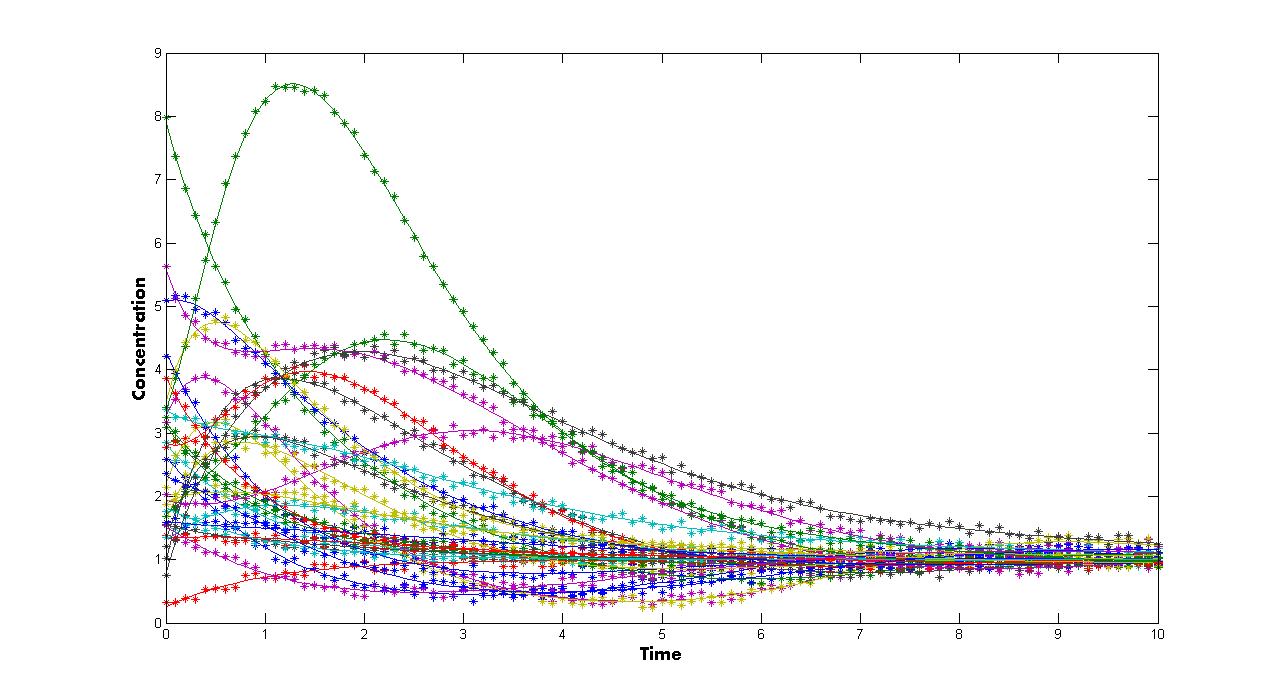


Figure 2 – 30-dimensional system result. A white noise with 10% variance was added to the time series. Then the signals were filtered using the Whittaker’s filter [4] and the parameter optimization was performed.

Eigenvalues of the Hessian Matrix

If defined a cost function *C* that quantifies the variation on the first derivative with variation in the parameter set (equation 15 in the main manuscript), the Hessian matrix can be calculate as

$$H\left(\delta\right)=\left.\frac{\partial^{2}C}{\partial\delta_{i}\partial\delta_{j}}\right|_{\delta=\delta^{*}}$$

where δ is the parameter vector. This matrix is can be used to study the model’s sensitivity to parameter variation. For computational experiments with real time series data used in this work, we calculate the Hessian matrix for each metabolite (decoupled form of the S-system) and its eigenvalues are shown in figure 2.


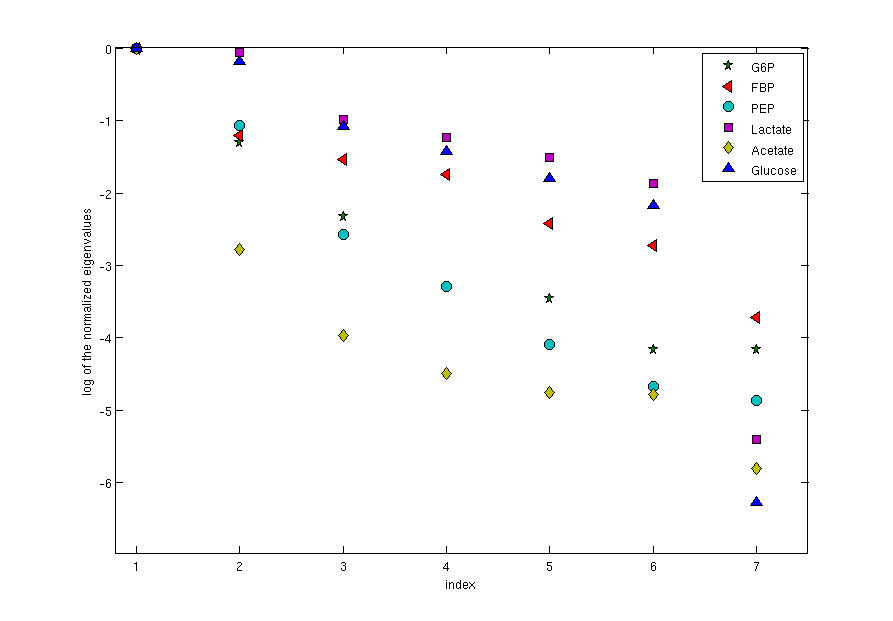


Figure 3 – Eigenvalues of the Hessian matrix normalized by the largest one. The eigenvalues are distributed in a large range (considerable difference between the largest one and the smallest one), suggesting that the dynamics of the system’s variation with respect to parameters are govern by few eigenvectors – stiff directions [5, 6].

Constraints on network topology

Here we show that the proposed optimization algorithm is flexible enough to impose a pre-specific network topology during the optimization process. This strategy is possible by removing from the following mapping $\psi\to Vp_{m} and \delta\to Vd_{m}$

$\begin{matrix} \log\left(\alpha_{m}\prod_{j=1}^{M} X_{j}^{h_{mj}}\right)=EigS\cdot\psi\\ \log\left(\beta_{m}\prod_{j=1}^{M} X_{j}^{g_{mj}}\right)=EigS\cdot\delta\end{matrix}$ 4

the metabolites (on the left hand side of equation 4) that do not interact in the production and/or degradation of a specific metabolite *m*. All the elements of the system above (equation 4) are defined in the main manuscript. To explore this idea, we use the *Lactococcus lactis* time series described in the main manuscript. Again, for modeling purposes, the concentrations of the metabolites have the following labels: glucose - *X1*; glucose 6-phosphate (G6P) - *X2*; fructose 1, 6-biphosphate (FBP) - *X3*; phosphoenolpyruvate (PEP) - *X4*; lactate - *X5*; acetate - *X6*. One of the systems found is shown in equation 5

. 5

The above system could be used as initial values of the MC process further analysis of its structure and parameter uncertainties, as suggest in the main manuscript.

Scripts description

This section describes the MATLAB® script used in all presented results.

Result=EO_mainf (TS,S,paramet)

Intput:

*TS_tp x m_* -time series of the state variables (tp – time points ; m –number of metabolites or state variables )

*S_tp x m_*- Slopes of the state variables

*paramet* – structure variable with the following fields

*paramet.iter* - number of iterations for the optimization algorithm – default =300;

*paramet.ubfi* - up boundary value of the linear combination vector – default=600;

*paramet.lbfi* - low boundary value of the linear combination vector – default=-600;

*paramet.ubB* - up boundary value of the constant rate Beta – default=300;

*paramet.ubk* - low boundary value of the kinetic parameters h – default=3;

*paramet.lbk* - low boundary value of the kinetic parameters h – default=-3;

*paramet.G* – Matrix where the element M_ij_ =1 if the metabolite j is present in the production term of the metabolite *i* and M_ij_ =0 otherwise – default = all elements 1;

*paramet.H* – Matrix where the element M_ij_ =1 if the metabolite j is present in the degradation term of the metabolite *i* and M_ij_ =0 otherwise – default = all elements 1;

paramet.A – vector of where each element v_i_=1 if the production term is present in the *ith* state variable equation;

paramet.B – vector of where each element v_i_=1 if the degradation term is present in the *ith* state variable equation.

paramet.int – scalar used to calculate the initial values for the optimization algorithm. It can be any positive number - default =1.

If neglected, the structure variable *paramet* will assume its default values.

Output

Result.Alfa

Result.g

Result.Beta

Result.h

Result.error

See supplementary material 2 for an example of how to use the functions (website.html) and the m-functions.

References

1. Voit EO, Almeida J: **Decoupling dynamical systems for pathway identification from metabolic profiles**. *Bioinformatics* 2004, **20**(11):1670-1681.

2. Kikuchi S, Tominaga D, Arita M, Takahashi K, Tomita M: **Dynamic modeling of genetic networks using genetic algorithm and S-system**. *Bioinformatics* 2003, **19**(5):643-650.

3. Kimura S, Ide K, Kashihara A, Kano M, Hatakeyama M, Masui R, Nakagawa N, Yokoyama S, Kuramitsu S, Konagaya A: **Inference of S-system models of genetic networks using a cooperative coevolutionary algorithm**. *Bioinformatics* 2005, **21**(7):1154-1163.

4. Vilela M, Borges CC, Vinga S, Vasconcelos AT, Santos H, Voit EO, Almeida JS: **Automated smoother for the numerical decoupling of dynamics models**. *BMC Bioinformatics* 2007, **8**:305.

5. Gutenkunst RN, Waterfall JJ, Casey FP, Brown KS, Myers CR, Sethna JP: **Universally sloppy parameter sensitivities in systems biology models**. *PLoS Comput Biol* 2007, **3**(10):1871-1878.

6. Gutenkunst RN, Casey FP, Waterfall JJ, Myers CR, Sethna JP: **Extracting falsifiable predictions from sloppy models**. *Ann N Y Acad Sci* 2007, **1115**:203-211.
