# Supplementary material for: Identification of neutral biochemical network models from time series data
Source: BMC Syst Biol. 2009 May 5;3:47. doi: 10.1186/1752-0509-3-47 (PMC2694766; doi:10.1186/1752-0509-3-47)
Supplement: Additional file 2 — Optimization algorithm implementation. This additional file provide the Matlab scripts of the optimization algorithm proposed in the main text. [file 1752-0509-3-47-S2.zip › webpage.html]

webpage


 


```
%Example1

F=load('example_ts.mat');%load a filtered time series file - [time_column metabolite1 2 ... N]
TS=F.TS;
der=central_diff(TS(:,2:end),TS(:,1));%time series derivative(time_series,time_points)
%G(i,j) is 1 if the metabolite j appears in the production term of
%metabolite i, otherwise G(i,j) is 0
paramet.G=[0 0 0 0 0 0;...
           1 0 0 0 0 0;...
           0 1 1 0 0 0;...
           0 0 1 0 0 0;...
           0 0 0 1 1 0;...
           0 0 0 1 0 0];

%H(i,j) is 1 if the metabolite j appears in the degradation term of
%metabolite i, otherwise H(i,j) is 0
paramet.H=[1 0 0 1 0 0;...
           0 1 0 0 0 0;...
           0 0 1 0 0 0;...
           1 0 0 1 0 0;...
           0 0 0 0 0 0;...
           0 0 0 0 0 0];
%vector A(i) is 1 if the production term is present in the equation of
%metabolite i and A(i) is 0 otherwise
paramet.A=[1 1 1 1 1 1];
%vector B(i) is 1 if the degradation term is present in the equation of
%metabolite i and B(i) is 0 otherwise
paramet.B=[1 1 1 1 1 0];

result=EO_mainf(TS(:,2:end),der,paramet);%Optimization function(time_series,first_derivative,opt_parameters)
%The opt_parameters will be set to its default values if not specified

for k=1:length(paramet.A)%for each metabolite
    Alfa(k)=result(k).alfa;
    Betta(k)=result(k).beta;
    g(k,:)=result(k).g;
    h(k,:)=result(k).h;
    error(k)=result(k).error;
end
Alfa
g
Betta
h
msgbox('End of optimization...Beginning of the integration')

x0=TS(1,2:end);%initial conditions
timeI=0:0.5:50;%integration time
options=odeset('Jacobian', @(t,x) Ssystem_jacobian(t,x,Alfa,g,Betta,h));%Jacobian matrix
[TI,XI] = ode23s( @(t,x) SsystemSym(t,x,Alfa,g,Betta,h),timeI,x0, options );%integration function
figure
plot(TI,XI)
hold on
F1=load('example_target.mat');%row data
MET=F1.MET;
plot(MET(:,1),MET(:,2:end),'o')
```

```
Alfa =

    1.6142    3.4072   11.7963    8.8794   14.2924    0.5449


g =

         0         0         0         0         0         0
    0.5162         0         0         0         0         0
         0    0.6113   -0.0786         0         0         0
         0         0    0.0402         0         0         0
         0         0         0   -0.2178   -0.0359         0
         0         0         0   -1.0582         0         0


Betta =

    4.1639    4.3470    6.2103    8.7407    7.7711         0


h =

    0.1219         0         0   -0.2978         0         0
         0    0.6439         0         0         0         0
         0         0    0.2788         0         0         0
    0.1346         0         0    0.1083         0         0
         0         0         0         0         0         0
         0         0         0         0         0         0
```

Published with MATLAB® 7.0
