# Supplementary figures and images for: Identification of neutral biochemical network models from time series data
Source: BMC Syst Biol. 2009 May 5;3:47. doi: 10.1186/1752-0509-3-47 (PMC2694766; doi:10.1186/1752-0509-3-47)

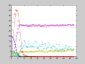

Supplement: Additional file 2 — Optimization algorithm implementation. This additional file provide the Matlab scripts of the optimization algorithm proposed in the main text. [file 1752-0509-3-47-S2.zip › webpage.png]

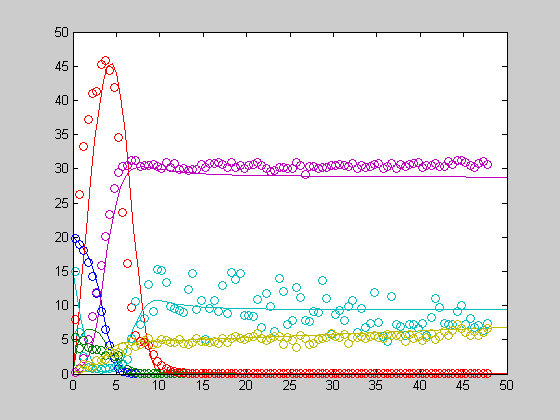

Supplement: Additional file 2 — Optimization algorithm implementation. This additional file provide the Matlab scripts of the optimization algorithm proposed in the main text. [file 1752-0509-3-47-S2.zip › webpage_01.png]
